# A novel approach to the program evaluation committee

**DOI:** 10.1186/s12909-019-1899-x

**Published:** 2019-12-16

**Authors:** Amy R. Schwartz, Mark D. Siegel, Alfred Ian Lee

**Affiliations:** 1 Yale University School of Medicine, 333 Cedar St, New Haven, CT 06510 USA; 2 VA Connecticut Healthcare System, Primary Care, Firm B, 950 Campbell Avenue, West Haven, CT 06516 USA

**Keywords:** Program evaluation, Medical education – graduate, Medical education – qualitative methods, American council of graduate medical education

## Abstract

**Background:**

The Accreditation Council for Graduate Medical Education requires each residency program to have a Program Evaluation Committee (PEC) but does not specify how the PEC should be designed. We sought to develop a PEC that promotes resident leadership and provides actionable feedback.

**Methods:**

Participants were residents and faculty in the Traditional Internal Medicine residency program at Yale School of Medicine (YSM). One resident and one faculty member facilitated a 1-h structured group discussion to obtain resident feedback on each rotation. PEC co-facilitators summarized the feedback in written form, then met with faculty Firm Chiefs overseeing each rotation and with residency program leadership to discuss feedback and generate action plans. This PEC process was implemented in all inpatient and outpatient rotations over a 4-year period. Upon conclusion of the second and fourth years of the PEC initiative, surveys were sent to faculty Firm Chiefs to assess their perceptions regarding the utility of the PEC format in comparison to other, more traditional forms of programmatic feedback. PEC residents and faculty were also surveyed about their experiences as PEC participants.

**Results:**

The PEC process identified many common themes across inpatient and ambulatory rotations. Positives included a high caliber of teaching by faculty, highly diverse and educational patient care experiences, and a strong emphasis on interdisciplinary care. Areas for improvement included educational curricula on various rotations, interactions between medical and non-medical services, technological issues, and workflow problems. In survey assessments, PEC members viewed the PEC process as a rewarding mentorship experience that provided residents with an opportunity to engage in quality improvement and improve facilitation skills. Firm chiefs were more likely to review and make rotation changes in response to PEC feedback than to traditional written resident evaluations but preferred to receive both forms of feedback rather than either alone

**Conclusions:**

The PEC process at YSM has transformed our program’s approach to feedback delivery by engaging residents in the feedback process and providing them with mentored quality improvement and leadership experiences while generating actionable feedback for program-wide change. This has led to PEC groups evaluating additional aspects of residency education.

## Background

The Accreditation Council for Graduate Medical Education (ACGME) defines program evaluation as the “Systematic collection and analysis of information related to the design, implementation, and outcomes of a graduate medical education program for the purpose of monitoring and improving the quality and effectiveness of the program.” [1] Historical models of medical education program evaluation assumed a linear cause-and-effect relationship between program components and outcomes, with a focus on measuring predetermined outcomes. Emphasis was placed on summative evaluation to determine intervention effectiveness [2]. However, graduate medical education occurs in a complex system, in which much learning is unplanned and curricula are only one part of a dynamic program context. Graduate medical education is affected by a myriad of factors internal and external to the program, including interdisciplinary relationships, patient factors, hospital infrastructure and policies, and resource constraints.

Newer program evaluation models suggest that evaluations should capture both intended and unintended (emergent) effects of medical education programs. They emphasize looking beyond outcomes, to understanding how and why educational interventions do or do not work [2]. In an internal medicine residency context, this might include understanding both anticipated and unanticipated effects of rotation elements on learning, workflows, faculty-resident relationships, morale, and the hidden curriculum. Program evaluation can also support a focus on ongoing formative feedback to generate actionable information towards program improvement [2–4]. In this way, the development and evaluation processes are intertwined and interdependent, promoting continuous adaptation to evolving context and needs.

There are a number of useful theoretical classifications of program evaluation. Kirkpatrick’s [5] four-level evaluation model parses program evaluation into [1] trainee reaction to the program, [2] trainee learning related to the program, [3] effects on trainee behavior, and [4] the program’s final results in a larger context. The CIPP (Context, Input, Process, and Project) evaluation model [6] divides program evaluation into four domains, [1] context evaluation, in which learner needs and structural resources are assessed, [2] input evaluation, which assesses feasibility, [3] process evaluation, which assesses implementation, and [4] product evaluation, which assesses intended and unintended outcomes. The CIPP model can be used for both formative and summative evaluations, and embraces the understanding of both predetermined outcomes and unplanned effects.

Musick [7] presents a useful, practical conceptual model of program evaluation for graduate medical education. The task-oriented model identifies five concrete steps to plan and carry out program evaluation. The first and second steps are to determine the rationale for evaluation and identify the specific entity to be evaluated. The third step involves specifying the evaluation methodology to collect and analyze data. The fourth step consists of determining to who and how results should be presented. In the fifth step, decisions are made regarding documentation of evaluation results.

Since 2014, the ACGME has required that each residency program have a Program Evaluation Committee (PEC) [8]. According to the ACGME, the PEC should participate in “planning, developing, implementing, and evaluating educational activities of the program,” “reviewing and making recommendations for revision of competency-based curriculum goals and objectives,” “addressing areas of non-compliance with ACGME standards,” and “reviewing the program annually using evaluations of faculty, residents, and others”. The program director appoints the PEC members, which must include at least one resident and two faculty. The PEC should utilize feedback from residents and faculty in the program-at-large to improve the training program and generate a written Annual Program Evaluation (APE).

The ACGME has few stipulations regarding how the PEC should carry out its duties. Several institutions have reported on data sources and structure for the APE or similar annual reviews [9–11]. However, there is little published peer-reviewed literature regarding how training programs have designed their PECs [12, 13]. In one published report, a general surgery program’s PEC met biannually and included 1 resident, faculty members, and program leadership [12]. Data reviewed by the PEC included surveys, resident exam performance, and clinical competency committee metrics. In another report, a psychiatry program’s PEC met every other week and examined feedback generated from monthly faculty advisor meetings, residency-wide meetings, resident evaluations, surveys, and a suggestion box, with action plans formulated by the PEC and other workgroups [13].

Historically, the Traditional Internal Medicine residency program at Yale School of Medicine (YSM) utilized an ACGME resident survey and online rotation evaluations as the primary means of soliciting feedback. While comprehensive, the evaluations were perceived by core faculty and residents as being difficult to interpret and not amenable to identifying actionable items for change. In response, the YSM Traditional Internal Medicine residency program designed an innovative approach to the PEC in which residents would have an active, meaningful role in programmatic evaluation. We hypothesized that structured resident group meetings would promote the synthesis of resident feedback and actionable suggestions, identifying key areas for programmatic improvement and offering trainees opportunities to be mentored in soliciting and giving feedback [14].

The Joint Committee on Standards for Educational Evaluation [15], a coalition of North American professional associations, has developed quality standards for the evaluation of educational programs. These encompass utility, feasibility, propriety, accuracy, and accountability standards. Utility standards are intended to maximize value to stakeholders; feasibility standards to increase effectiveness and efficiency; propriety standards to support fairness and justice; accuracy standards to maximize evaluation reliability and validity; and accountability standards address documentation and evaluation of the evaluation process itself.

In developing our PEC, we sought to emphasize the utility standards of evaluator credibility, attention to stakeholders, negotiated purpose, and timely and appropriate communicating and reporting. We also sought to affirm in particular the feasibility standard of practical procedures, the propriety standard of transparency and disclosure, and the accountability standard of internal meta-evaluation.

## Methods

### Research design

We devised and implemented a novel structure for the YSM Traditional Internal Medicine residency PEC, in which residents led structured group discussions involving other residents, with the purpose of evaluating each of the inpatient and outpatient teaching firms in our training program. Over a 4-year period, we compiled actionable feedback arising out of the PEC focus group discussions, and we surveyed PEC residents and teaching faculty to assess perceptions of the PEC process.

### Setting and participants

The YSM Traditional Internal Medicine residency program consists of 124 internal medicine and 14 preliminary residents. Inpatient and outpatient rotations are divided into firms, each overseen by a member of the core teaching faculty who is given a designated title of “Firm Chief.” Most rotations occur on two campuses: Yale-New Haven Hospital (YNHH) and the Veterans Affairs (VA) Connecticut Healthcare System. Seventeen rotations are reviewed by the PEC: 10 inpatient firms at YNHH, three inpatient firms at the VA, and four outpatient clinics.

### Program description

**Fig. 1:**
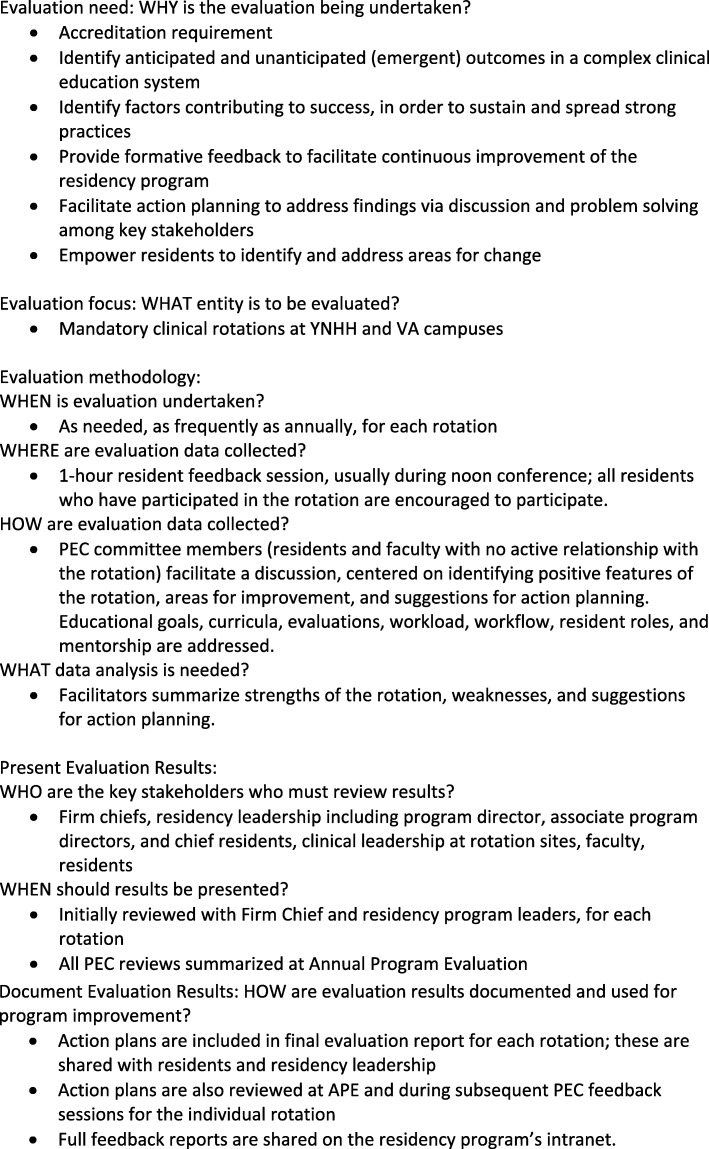
Task-Oriented Conceptual Model of the YSM Traditional Internal Medicine Residency PEC

A Task-Oriented Conceptual Model [7] of the YSM Traditional Internal Medicine Residency PEC is shown in Fig. 1. Feedback is obtained from resident group discussions facilitated by residents and faculty who serve on the PEC. All residents in the Traditional Internal Medicine residency program and preliminary interns are invited to join the YNHH or the VA PEC as committee members. At the start of each academic year, the PEC determines which rotations require review for the year, ensuring that all rotations are reviewed on a regular basis and that rotations undergoing or in need of change are reviewed. During the first year of the PEC initiative, all inpatient and outpatient rotations at YNHH and the VA were designated for formal review by the PEC; rotations in which the PEC process identified deficits requiring systematic change were then selected for repeat review by the PEC over subsequent years. The total follow-up period for this study is 4 years.

Each rotation is assigned a resident-faculty pair in which a PEC resident evaluates the rotation under supervision by a designated faculty member. In every instance, PEC faculty facilitators assigned to review a specific rotation have no active relationship with that rotation. A 1-h group resident feedback session is held for each rotation, generally during the noon conference time; all residents are encouraged to participate. The assigned PEC resident is responsible for facilitating the discussion utilizing a structured format centered on four questions:

1. What are the positive features of this rotation?
2. What are areas for improvement?
3. What features should be adopted on other rotations?
4. What aspects should be changed, and how?

Prior to the group discussion, the PEC resident assigned to review the rotation is encouraged to engage with the appropriate Firm Chief to identify specific areas to focus on for feedback. During the group discussion, the PEC facilitators ensure that educational goals, curricula, evaluations, workload, workflow, resident roles, and mentorship are addressed, and that resident anonymity is preserved. After the discussion, the PEC facilitators compile additional email feedback from residents who were unable to attend the group discussion, summarize the feedback in a written document, and meet with the Firm Chief in addition to other residency program leadership and teaching faculty to deliver the feedback. The PEC facilitators, Firm Chief, and program leadership together develop an action plan, and the written feedback is submitted to the program and reviewed at the APE.

### Data collection

We collected two types of data during the 4-year study period: [1] summative evaluations of individual inpatient and outpatient rotations generated by the PEC process, and [2] resident and faculty perceptions of the PEC process. Summative evaluations of the different rotations were obtained from the written documents created by the PEC residents upon completing their evaluations of individual firms, and from the action plans created by the PEC facilitators, Firm Chiefs, and program leadership. Resident and faculty perceptions of the PEC process were gauged using surveys.

### Survey design

We sought to understand how feedback obtained by the PEC process was perceived and acted upon by faculty Firm Chiefs in comparison to written surveys. We also wanted to understand how residents and faculty participating on the PEC perceived the PEC process and whether such participation had an impact on resident/faculty mentorship, delivering feedback, or facilitating small group discussions. To assess for these factors, the PEC faculty designed two surveys, one for faculty Firm Chiefs, the other for PEC residents and faculty, aimed at addressing these questions. Survey questions were proposed, drafted, discussed, and edited by faculty members of the PEC. Content validity of the surveys was assessed by one coauthor (M.D.S.) who was involved in the design and implementation of the PEC process but not in the initial generation of survey questions. The surveys were tested on 3 YSM faculty and further modified according to their feedback, with the revised surveys incorporating simplified “Yes/No” response options and multiple prompts for free text responses. The final surveys were distributed electronically using SurveyMonkey to all faculty Firm Chiefs and PEC faculty and resident members in 2016 and 2018 after completion of the second and fourth years, respectively, of the project [16].

### Data analysis

For each inpatient and outpatient rotation, major recommendations were compiled from the written documents created by PEC residents and from the action plans created by PEC facilitators, Firm Chiefs, and program leadership. For the 2016 and 2018 surveys of PEC residents and Firm Chiefs, response rates and responses to “Yes/No” and free-text questions were analyzed. The small sample size of the study population precluded statistical analysis of any answers to survey questions. The free text responses for both surveys were independently reviewed by two authors (ARS and AIL) according to Miles and Huberman [17]; themes emerging from these responses were identified and analyzed for repetitive patterns and contrasts. The two investigators then met to discuss the themes; at least 80% agreement on themes was achieved, with discrepancies resolved by consensus discussion. The exact survey instruments are shown in Additional file 1 (survey of faculty Firm Chiefs) and Additional file 2 (survey of PEC member residents and faculty).

## Results

During the course of four academic years (2014–15 through 2017–18), 40 residents and 14 faculty (including chief residents) participated as PEC facilitators.

### PEC findings

Over the 4-year study period, the PEC process generated a number of specific points of feedback on every inpatient and outpatient rotation. At both the YNHH and VA campuses, numerous services were lauded for their outstanding educational experiences and other positive attributes. Many suggestions for change were able to be implemented over subsequent years. Summaries of positive features, recommended changes, and feedback implementation after the first year of the PEC initiative are shown in Table 1 (YNHH) and Table 2 (VA Hospital).

**Table 1:** Summary of PEC findings on inpatient and outpatient rotations at YNHH (year 1)

Rotation	Positive features	Suggestions for change
Donaldson Service (Infectious Disease)	Outstanding attendings	• Need 2 attendings on service at all times
Peters Service (Nephrology)	Outstanding educational experience	• Improve weekend schedule • Hire physician extender
Fitkin Service (General Medicine)	Outstanding experience overall	• Designate teaching cases for admission to Fitkin while patients are still in the emergency department
Generalist (General Medicine)	Unique patient population and curriculum	• Optimize daily call schedule and workflow
Cardiac ICU	Outstanding educational experience	• Optimize daily workflow and patient flow
Goodyer Service (Cardiology)	Lighter workload than other busier services allowing for deeper intellectual immersion	• Redesign team structure to better balance out work responsibilities on other busier services
Hematology	Outstanding educational experience	• Need 2 residents on service
Oncology	Outstanding educational experience	• Need 2 residents on service • Clarify resident role in goals-of-care discussions
Primary Care Clinic	Outstanding ambulatory medicine experience	• Logistical and workflow problems

**Table 2:** Summary of PEC findings on inpatient and outpatient rotations at the VA (year 1)

Rotation	Positive features	Suggestions for change
Inpatient wards	• High-yield teaching and rounds • Interesting patient population • Good autonomy	• Standardize transition of patients from the emergency department to the floor • Optimize the high workload of admitting days • Optimize ways to update nurses on rounds
MICU	• One-on-one educational experience • Intimate collaboration with colleagues	• Optimize flow process for nighttime radiology • Address conflicts of understanding regarding VA policy
Medicine consults	• Good resident autonomy • Good variety of cases • Appropriate patient volume • Opportunity for residents to learn how to be a consultant	• Make curriculum widely available • Increase teaching experiences
Firm A and B clinics	• Excellent precepting by attendings • High quality of care with enjoyable patient population	• Clarify expectations • Involve residents more meaningfully in teamwork
Women’s clinic	• Outstanding attendings • Good exposure to women’s health • Balanced workload • Excellent half-day teaching session	• Improve formal curriculum
Center of Excellence	• Favorable team structure • Good mix of didactics and patient care	• Optimize patient workflow • Increase opportunities for procedures

While many of the PEC’s findings were unique to individual rotations, several common themes emerged in PEC feedback reports for multiple rotations. Frequently cited strengths included:

- Caliber of teaching. Residents described a high caliber of precepting and teaching by ward and clinic attendings on the majority of inpatient and outpatient rotations. Examples include, “quality of attending rounds is phenomenal, caters to resident, intern, and med student levels,” “the VA MICU is a great place to get to know your attending very well, develop mentors, get career advice, and get very specific, day to day feedback,” “attendings at the VA are amazing life mentors … and are very optimistic with strong passion and enthusiasm for work that is contagious,” and “both women’s clinic preceptors were excellent mentors, approachable, very good teachers.”
- Patient care. Residents found their inpatient and ambulatory patient care experiences to be highly diverse and of great educational value. Examples include, “the high clinical volume, complicated patients, and new patient evaluations were noted as maximizing educational value on this rotation,” “enjoyed having the experience of being a consultant,” “amazing VA patient population … it is quite an honor to serve them and to learn specific military and exposure histories,” and “sufficient diversity of diseases … many bread and butter cases as well as multiple different specialty cases.”
- Interdisciplinary care. Residents encountered a strong emphasis on interdisciplinary care across many services, which improved the quality of care delivered. Examples include, “incorporating other clinicians (APRNs, pharmacists, health psych [ology]) into daily work flow helps with management of difficult patients,” “exceptionally comprehensive multidisciplinary set-up,” “case management rounds are very efficient,” and “nurses are helpful, informative.”

Common areas for improvement included:

- Didactics. On several rotations, residents identified a need to optimize the orientation process and to streamline or improve availability of educational curricula. For example, “residents are unclear of the goals for specific immersion blocks,” “there is currently no curriculum to guide independent study. Attending rounds have not been a feature of the rotation,” “would like more women’s health didactics during educational half days,” and “some teams sat down and defined their learning goals and career interests the first day of the rotation … it would be great if this process became standard for all teams.”
- Interactions with other services. Residents identified needs for improvement in communication with nursing staff, utilization of specialty services, and navigation of disposition decisions between services. For example, “very difficult to obtain imaging studies overnight,” “medicine appears to be the default service to admit to … there is a concern that some patients could benefit and be managed better from being on the specialty service,” and “very difficult to reach [several services].”
- Technological issues. Residents found problems with the paging system and with computer or identification badge access on some rotations. For example, “residents proposed making access easier to the Psychiatry ER and [psychiatry] inpatient units, such as by granting this access on their VA identification card,” “some residents reported consistent glitches in home access,” “sometimes the pagers do not work due to dead zones. It would be helpful to move to a phone or otherwise more reliable electronic system,” and “[confusing] charting of ED medications.”
- Workflow. Residents identified problems with call schedules, admitting algorithms, and balance between teaching and service on certain rotations. Examples include, “clinic work flow is inefficient … .geographic barriers (clinic is very far from check in and the waiting room) and poor staffing,” “if patients are already signed out to be admitted to the floor but are still physically present in the ED … .residents found it challenging to manage … because of the distance between the medicine floors and the ED,” “imbalanced admission loads on different admitting days due to diversion,” and “social admissions and observation patients accounted for a large number of their census.”

### Evaluation of the PEC

**Fig. 2:**
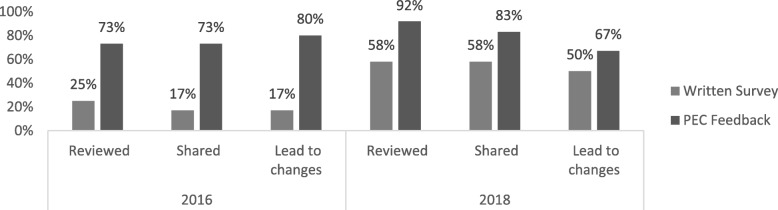
Comparison of written surveys vs. PEC feedback by Firm Chiefs in 2016 and 2018. For 2016, a greater number of Firm Chiefs reviewed, shared, and responded to feedback generated by the PEC than by written surveys; all of these differences were statistically significant (*p* = 0.04, 0.01, and 0.01, respectively). For 2018, a greater number of Firm Chiefs reviewed, shared, and responded to PEC vs. written survey data but these findings were not statistically significant (*p* = 0.16, 0.37, and 0.40, respectively)

To understand how feedback obtained by the PEC was viewed and utilized, we designed a survey, sent to all Firm Chiefs, which asked whether PEC feedback was reviewed and shared with other faculty or administrators, and whether the PEC process led to changes in the rotation. The same questions were asked about traditional written evaluations filled out by residents. Surveys were sent to a total of 16 Firm Chiefs in 2016 and 17 in 2018; response rates for each survey were 75 and 71%, respectively. As shown in Fig. 2, in both years, Firm Chiefs reviewed, shared, and responded with change to feedback obtained from the PEC with greater frequency than that obtained from written resident evaluations. Firm Chiefs preferred to receive feedback from both the PEC and written evaluations rather than only the PEC or only written evaluations alone (for 2016: 90.9, 9.1, and 0%, respectively; for 2018: 50, 33, and 17%, respectively).

To understand how PEC participants viewed their experiences participating in and contributing to the PEC process, we also designed a survey, sent to all resident and faculty PEC members, which asked about their perspectives on the utility of the PEC process and whether participation in the PEC led to resident-faculty mentoring relationships and improvement in skills of facilitating and delivering feedback. Surveys were sent to a total of 26 PEC members in 2016 and 25 in 2018; response rates for each survey were 69 and 80%, respectively. Across both years, 84% of survey respondents indicated that their experiences with the PEC improved their feedback skills. The majority of respondents developed a mentoring relationship as a result of the PEC. Committee members were also asked to provide written commentary regarding the feedback process; major themes as identified using content analysis are shown in Table 3 (with representative comments):

**Table 3:** Committee member perceptions of the PEC feedback process

Theme	Examples
PEC feedback is actionable	“[The PEC] allows for direct feedback that in some ways has a greater potential to create change as it represents the views of the aggregate group.”
	“Unlike other mechanisms of feedback, the PEC is truly a bi-directional discussion that ultimately focuses on modifiable elements.”
	“What is added [by the PEC] is an authenticity and summation of feedback with the opportunity to discuss with leadership constructively to develop a plan for moving forward.”
Open discussion provides for more effective feedback generation than written surveys	“[The PEC is] Very helpful. Allows for open and critical review of rotations. Surveys will tend to get discarded and only those polarized to opposite ends may respond (i.e. only those very happy or very unhappy). With live feedback we can get people of all different viewpoints.”
	“I think [the PEC] is less biased than other program feedback mechanisms because the feedback is anonymous and critically evaluates the program.”
	“Direct feedback in a group setting allows the residents to discuss and build consensus on faculty “best practices” as well as issues that are most important to them.”
	“The main benefit [of the PEC] is that it is done in a live, town hall style session where residents are able to give specific feedback as opposed to surveys that are often disregarded and filled out as quickly as possible.”
The PEC is a great learning opportunity	“It gives residents the unique opportunity to get involved in the feedback process and learn to facilitate a focus group as well as learn to pare down tons of suggestions and information into a concise report.”
	“In resident feedback sessions I have learned how to redirect conversation while still making sure people are heard. It is also a new skill to provide constructive criticism and feedback to my superiors.”
	“I think that the process of trying to be an unbiased facilitator at the meetings is a valuable experience. Then, after the feedback sessions, I can synthesize the feedback in a way that I hope will lead to a productive meeting.”

### Expansion of the PEC

As a result of the PEC’s ability to identify actionable feedback for change, in the years following the PEC’s inception, residents in the Traditional Internal Medicine program at YSM adopted the PEC format not only for continued assessment and optimization of inpatient and outpatient firms but also for examination of a number of broad residency domains such as educational conferences, call structures, and evaluations. During the 2015–2016 academic year, several residents created a PEC subgroup focused on medical education, which used structured group discussions and surveys to identify several areas for potential change; as a result of this effort, a noon conference committee was convened in the 2018–2019 academic year that completely redesigned the noon conference series for the year. During the 2018–2019 academic year, the PEC group again utilized structured group discussions and surveys to evaluate resident perceptions of hospital rotations with a 28-h call schedule, leading to the creation of a 28-h call working group to restructure those services. PEC residents also undertook formal evaluations of the residency program’s leadership (including chief residents, associate program directors, and the program director) using the same PEC format for eliciting verbal group and written feedback.

Many residents also indicated a need for the PEC to better inform the program-at-large regarding PEC findings and action plans. In response to this, in 2018 the PEC began posting its feedback reports on our residency program’s intranet. Going forward, the PEC will also look to identify more robust processes for follow-up of action plans, and to align the PEC feedback process with other ongoing quality improvement initiatives (i.e. resident team quality improvement projects, chief resident for quality and safety interventions).

## Discussion

Over the course of four years, the PEC for the YSM Traditional Internal Medicine residency program transformed the process by which feedback is obtained from residents and delivered to educational leadership. We emphasized the role of residents in facilitating, synthesizing, and delivering feedback. Compared to written evaluations, feedback generated by the PEC was more likely to be reviewed and responded to by Firm Chiefs. Residents and faculty who participated in the PEC generally found that their experiences improved their feedback skills, and many participants developed resident-faculty mentoring relationships. The success of our approach to the PEC led to the development and implementation of additional projects to expand the PEC’s scope.

Firm Chiefs preferred to obtain feedback from both the PEC and written surveys rather than either one alone. Focus groups and written surveys are complementary tools; written surveys provide quantitative data, while group discussions provide qualitative data that allow for brainstorming, synthesis, and prioritization. Implementation of PEC discussion groups cannot entirely supplant surveys, as the ACGME requires that residents have the opportunity to evaluate the program anonymously in writing.

In the Firm Chief surveys, between 2016 and 2018, there was an apparent rise in the percentage of Firm Chiefs who made use of written surveys across the two years. It is possible that this may reflect an increase in awareness and/or accessibility of the results of the written surveys during this time period, although some of the differences may alternately reflect the small number of respondents. The 2016 and 2018 Firm Chief surveys also showed an interval decline in the percentage of Firm Chiefs who responded with change to feedback obtained from the PEC, which could be due to a diminishing need to launch systematic changes when actionable feedback is being regularly obtained.

Newer program evaluation models [2], including the CIPP model [6], focus on understanding the complex educational system, assessing unanticipated outcomes, and formative evaluation. Findings from our PEC rotation evaluations reflected this complexity in graduate medical education, in which learning and the trainee experience are affected by many factors internal and external to the program. We found that open-ended discussions focused on rotation strengths and areas for improvement allowed for reflection on unanticipated outcomes (i.e. communication issues with other services, lack of standardization of patient transitions, slow patient workflows, and imbalanced workloads), as well as those more directly related to the curriculum (i.e. need for improvements in orientation and curricula). This approach also facilitated identification of best practices and ideas for sustainment and spread. The emphasis on formative feedback allowed us to identify actionable items for change, conduct bi-directional discussions among stakeholders, and participate in the iterative improvement of the program.

Standards for evaluation of educational programs include the dimensions of utility, feasibility, propriety, accuracy, and accountability [15]. We sought to maximize utility via the values of evaluator credibility (residents and faculty both have an active leadership and facilitation role, PEC committee members have no relationship with the rotation that they review); attention to stakeholders (residents, faculty, program leadership, and firm chiefs are involved in assessment and action planning); negotiated purpose (discussion with firm chiefs regarding areas of desired feedback, focus on helping medical educators optimize education and clinical care); and timely and appropriate communicating and reporting (stakeholder meetings regarding action planning, written documentation of feedback and action plans, reporting via APE meeting, email, and intranet.) We sought to emphasize the feasibility standard of practical procedures (use of available conference time resources, meeting in locations easily accessible to residents). We asserted the propriety standard of transparency and disclosure by making full reports available to stakeholders. Our periodic structured inquiry to PEC members and firm chiefs regarding their experience and needs sought to satisfy the accountability standard of internal meta-evaluation.

There are several limitations to our study. We took a participant-oriented approach that sought to determine how trainees experienced their rotations [3]. However, this limited us to level 1 of Kirkpatrick’s program evaluation model [5], trainee reaction, and did not directly assess learning, behavior, or clinical outcomes. Ultimately, we found that our PEC approach was most effective when used as a supplement to many other measures considered at our APE, including board scores, fellowship and career information, and Faculty and Resident ACGME surveys. Another limitation is the focus on the experience of resident stakeholders; however, we made this choice due to a perceived need for resident input by program leadership. Faculty and other stakeholder input is also considered at both the APE and individual rotation action planning meetings. Additional limitations of our study include generalizability, as successful implementation at our program may reflect the institutional culture or other elements specific to our program; and the small numbers of Firm Chiefs and committee members in a single training program. Also, the feedback obtained during resident group discussions is dependent on the quality of the facilitation and level of resident engagement.

## Conclusions

In conclusion, we created an innovative format for the PEC, consisting of structured resident discussions led by resident-faculty facilitators. These are synthesized to generate actionable feedback for each rotation. Feedback is presented in person to Firm Chiefs, concrete action plans created, and residency and clinical leadership alerted to rotation strengths and areas for improvement. Our PEC experience has had a sustained and transformative impact on the feedback process for our residency program.

## Supplementary information

**Additional file 1.** 2018 PEC Survey – Firm Chiefs. Text of electronic survey to assess firm chief perceptions of PEC Feedback.

**Additional file 2.** 2018 PEC Survey – Committee Members. Text of electronic survey to assess committee member experiences on the PEC.

## Data Availability

The datasets used and/or analyzed during the current study are available from the corresponding author on reasonable request.

## Abbreviations

ACGME: Accreditation Council for Graduate Medical Education
APE: Annual Program Evaluation
CIPP: Context Input Process Product
PEC: Program Evaluation Committee
VA: Veterans Affairs
YNHH: Yale-New Haven Hospital
YSM: Yale School of Medicine
