# Supplementary material for: A novel approach to the program evaluation committee
Source: BMC Med Educ. 2019 Dec 16;19:465. doi: 10.1186/s12909-019-1899-x (PMC6916454; doi:10.1186/s12909-019-1899-x)
Supplement: Supplementary file 1 — Additional file 1. 2018 PEC Survey – Firm Chiefs. Text of electronic survey to assess firm chief perceptions of PEC Feedback. [file 12909_2019_1899_MOESM1_ESM.docx]

2018 PEC Survey - Firm Chiefs

Firm Chiefs receive feedback on their clinical rotation from (1) written surveys completed by housestaff on medhub and (2) the Program Evaluation Committee (PEC) resident focus group feedback session. We would like to better understand what feedback is valuable to you in shaping your clinical rotation, in order to optimize the feedback we give you. Please answer the following questions about the two feedback mechanisms:

1. Did you review the written survey this academic year?

Yes

No

2. Did you share the written survey results with other faculty or administrators?

Yes

No

3. Did the written survey lead to an action plan or changes in your clinical rotation?

Yes

No

If Yes, what was the action plan or change?

4. What is helpful about the written survey? What elements of the written survey could be improved?

Comments:

5. Did you review the PEC feedback this academic year?

Yes

No

6. Did you share the PEC feedback results with other faculty or administrators?

Yes

No

7. Did the PEC feedback lead to an action plan or changes in your clinical rotation?

Yes

No

Other (please specify)

8. What is helpful about the PEC feedback? What elements of the PEC feedback could be improved?

Comments:

9. Which would you prefer to receive in the future?

written survey

PEC feedback

Both the written survey and the PEC feedback

Would prefer some other type of feedback

Comments:

10. How could the residency program assist you with action planning for improvements/changes to your rotation?

Comments:

11. What other input do you have about resident feedback? How can we make this most helpful to you?

Comments:
