# Supplementary material for: A novel approach to the program evaluation committee
Source: BMC Med Educ. 2019 Dec 16;19:465. doi: 10.1186/s12909-019-1899-x (PMC6916454; doi:10.1186/s12909-019-1899-x)
Supplement: Supplementary file 2 — Additional file 2. 2018 PEC Survey – Committee Members. Text of electronic survey to assess committee member experiences on the PEC. [file 12909_2019_1899_MOESM2_ESM.docx]

2018 PEC Survey -- Committee Members

The Program Evaluation Committee (PEC) is completing its third year. We would like your feedback as a committee member, in order to make the committee as useful as possible for the residents, faculty, and residency program. Please answer the following questions:

1. Please indicate whether you are faculty or a resident

Faculty

Resident

2. Did you facilitate or co-facilitate a resident feedback session? (i.e. lead a session or writeup the session's feedback)

Yes

No

3. Is the PEC feedback process helpful for aiding faculty in optimizing the educational and clinical rotation experience? How does it differ from other residency program feedback mechanisms you have seen or been involved in?

Comments:

4. What is helpful in the PEC feedback process?

Comments:

5. What could be changed to optimize the PEC feedback process?

Comments:

6. For faculty: did participation in the committee enable you to mentor one or more residents?

For residents: did participation in the committee enable you to receive mentoring from faculty?

By "mentoring", we mean broadly a meaningful or valuable interaction that is distinct from your usual clinical relationships with faculty (if you are a resident) or residents (if you are faculty).

Comments:

7. Did participation in the committee improve your facilitation, mentoring, focus group , or delivering feedback skills?

Yes, a lot

Yes, a little

No

Please comment

8. What could we do to improve your experience on the PEC?

Comments:

9. What should we do to more effectively inform residents about the PEC findings?

Comments:
